# Supplementary material for: Synergistic Upregulation of Extracellular Vesicles and Cell-Free Nucleic Acids by Chloroquine and Temozolomide in Glioma Cell Cultures
Source: Int J Mol Sci. 2025 Oct 4;26(19):9692. doi: 10.3390/ijms26199692 (PMC12524867; doi:10.3390/ijms26199692)
Supplement: Supplementary file 1 [file ijms-26-09692-s001.zip › supplementary figure S1.pdf]

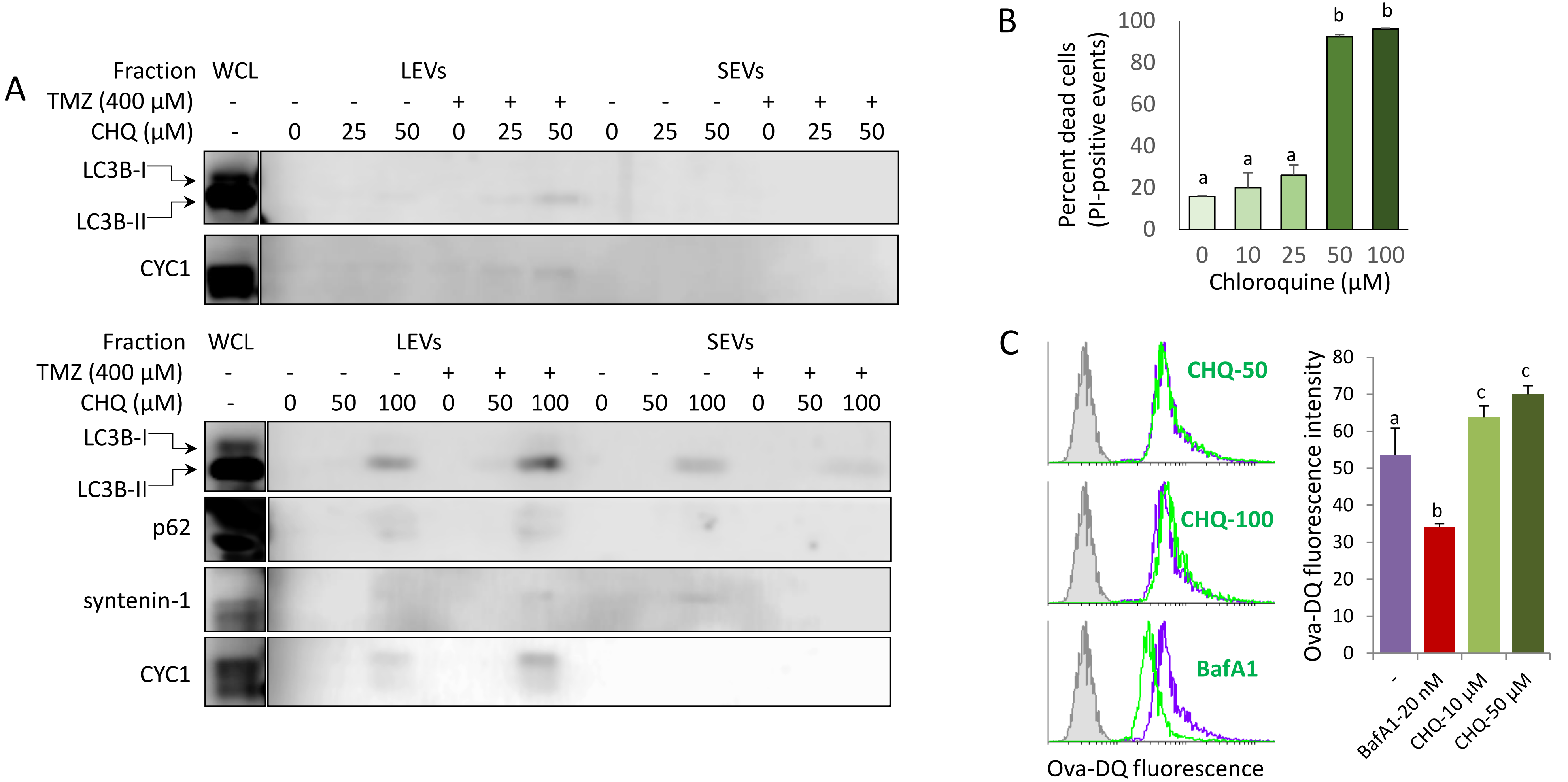

Supplementary figure S1. At high concentrations, CHQ induces secretion of EV and autophagy related markers, along with CYC1, which is further enhanced by TMZ and might result from the cytotoxicity of the treatment on THP-1 cells. (A) WB analysis of EV samples from two independent experiments, using the indicated concentrations of CHQ. (B) Cytotoxicity of treatments with different concentrations of CHQ for 48 h measured with PI staining and flow cytometry. (C) In contrast to BafA1, CHQ does not inhibit the hydrolysis of the fluorescent substrate Ova-DQ in THP-1 cells. The cells were pre-incubated with the indicated concentrations of inhibitors for 30 min, before the addition of 15 µg/ml of DQ™ Ovalbumin (Invitrogen). The fluorescence of the digested product (Ex/Em - 505/515 nm) was measured with flow cytometry after 24 h of incubation. The representative overlay histograms show the control w/o substrate (filled gray contour), the control cells incubated with DQ-Ova only (open purple contour), and the cells co-incubated with substrate and inhibitor as indicated (open green contour). Data was analyzed with one-way ANOVA; n=3; $p<0.05$. Although, it appears there might be a synergistic upregulation of secretory autophagy by TMZ and high concentrations of CHQ, the detected signals were very weak, and the results could not be confirmed in subsequent experiments. The high levels of cytotoxicity are likely accountable for the irreproducibility of the results, hampering any unequivocal conclusions.
