# Supplementary material for: Synergistic Upregulation of Extracellular Vesicles and Cell-Free Nucleic Acids by Chloroquine and Temozolomide in Glioma Cell Cultures
Source: Int J Mol Sci. 2025 Oct 4;26(19):9692. doi: 10.3390/ijms26199692 (PMC12524867; doi:10.3390/ijms26199692)
Supplement: Supplementary file 1 [file ijms-26-09692-s001.zip › supplementary figure S2.pdf]

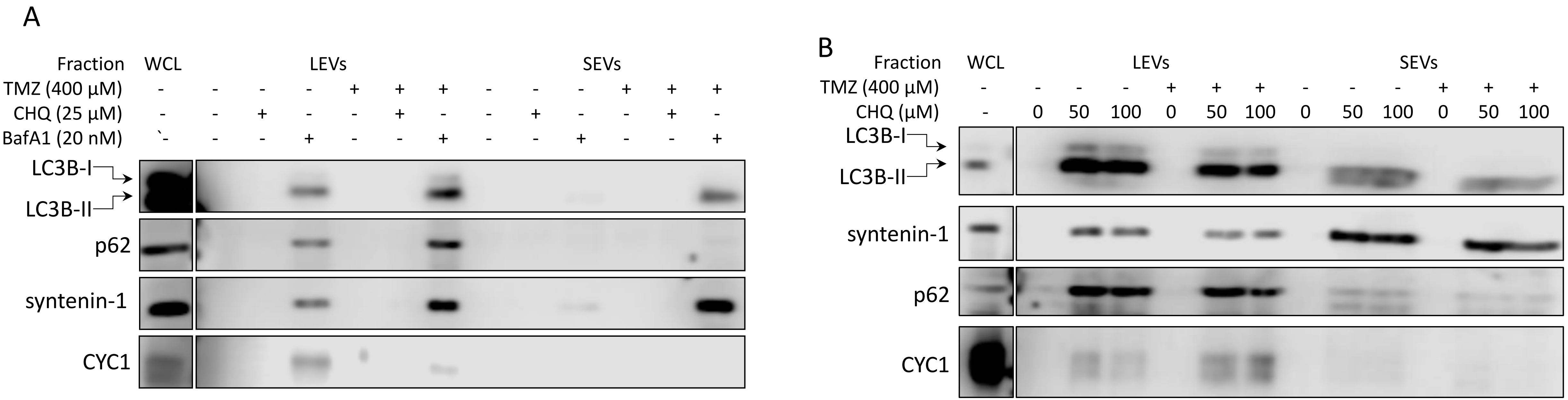


Supplementary figure S2. (A) Similarly to undifferentiated THP-1 monocytes, PMA-differentiated THP-1 macrophages respond to TMZ+BafA1 treatment with enhanced secretion of autophagy markers. (B) CHQ concentrations ≥ 50 µM upregulate release of EV and autophagy related markers, as well as CYC1. In accordance with an established protocol for generation of THP-1 macrophages (DOI: 10.3390/nano7100332 ), THP-1 cells were treated with 10 ng/ml of phorbol myristate acetate (PMA) for 24h, with a subsequent incubation in PMA-free medium for 72h . The cells were then treated as indicated for 48 h, and EVs were isolated and analyzed with WB, as described for the other experiments. The shown results are from two independent experiments.
