# Supplementary material for: Synergistic Upregulation of Extracellular Vesicles and Cell-Free Nucleic Acids by Chloroquine and Temozolomide in Glioma Cell Cultures
Source: Int J Mol Sci. 2025 Oct 4;26(19):9692. doi: 10.3390/ijms26199692 (PMC12524867; doi:10.3390/ijms26199692)
Supplement: Supplementary file 1 [file ijms-26-09692-s001.zip › supplementary figure S3.pdf]

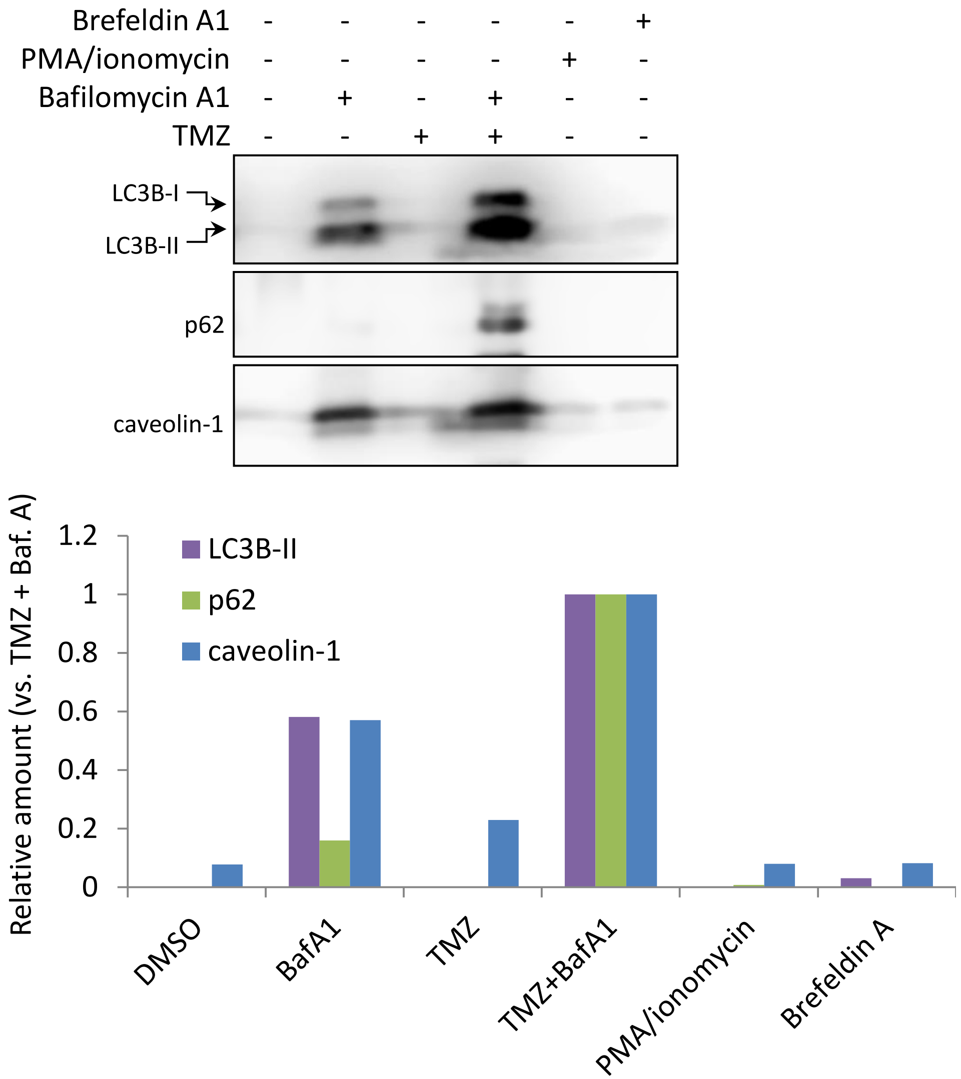

Supplementary figure S3. TMZ enhances the BafA1-induced secretion of LC3B and p62 by U138 cells. WB with LEV samples from U138 cells, treated with 400 µM TMZ and 50 µM BafA1 for 48 hours. The histogram beneath the blot images show the densitometry values. There was no significant upregulation of LC3B-II, p62 or caveolin-1 in LEV samples from cells, treated with 3 µM Brefeldin A1, or with a combination of 10 ng PMA and 1uM ionomycin – an apoptosis inducer in glioma cell cultures (doi: 10.1371/journal.pone.0076717).
