# Supplementary material for: Synergistic Upregulation of Extracellular Vesicles and Cell-Free Nucleic Acids by Chloroquine and Temozolomide in Glioma Cell Cultures
Source: Int J Mol Sci. 2025 Oct 4;26(19):9692. doi: 10.3390/ijms26199692 (PMC12524867; doi:10.3390/ijms26199692)
Supplement: Supplementary file 1 [file ijms-26-09692-s001.zip › Supplementary_Table_S1.pdf]

Supplementary Table S1. **Reported IC_{50} values of temozolomide (TMZ) and chloroquine (CHQ) in U87-MG and U138-MG glioma cell lines.**

**Cell line**	**Drug**	**Exposure time / assay**	**Reported IC50 (µM)**	**Reference**
U87-MG	Temozolomide	24 h, various assays (systematic review)	123.9 (median)	Poon et al., 2021 https://doi.org/10.1186/s12885-021-08972-5
U87-MG	Temozolomide	48 h, various assays (systematic review)	223.1 (median)	Poon et al., 2021 https://doi.org/10.1186/s12885-021-08972-5
U87-MG	Temozolomide	72 h, various assays (systematic review)	230.0 (median)	Poon et al., 2021 https://doi.org/10.1186/s12885-021-08972-5
U87-MG	Temozolomide	72 h, MTT assay	~200–400	Soni et al., Cancers 2021 https://doi.org/10.3390/cancers13174485
U138-MG	Temozolomide	72 h, CCK-8 assay	973.6	Liu et al., Mol Med Rep 2014 https://doi.org/10.3892/mmr.2014.2811
U87-MG	Chloroquine	72 h, TMZ+CHQ study (J Neurooncol)	10 µM used in combination; IC50 not specified	Hori et al., J Neurooncol 2015 https://doi.org/10.1007/s11060-014-1686-9
Glioma cell lines (general)	Chloroquine	48–72 h, multiple assays	20–50 (range)	Müller et al., Cells 2023 https://doi.org/10.3390/cells12091290

Notes: Reported IC_{50} values vary widely depending on assay type (MTT, CCK-8, LDH), serum content, exposure duration, and cell line passage/source. U87-MG is generally TMZ-sensitive ($IC_{50} \approx 100–300$ µM at 24–72 h), while U138-MG is more TMZ-resistant (IC_{50} often >900 µM). CHQ IC_{50}s are often in the tens of µM range, although 10 µM is frequently used to inhibit lysosomal function and autophagy.
